# Prevalence and underlying factors of mobile game addiction among university students in Bangladesh

**DOI:** 10.1017/gmh.2021.34

**Published:** 2021-09-06

**Authors:** Md Abu Sayeed, Md Shabbir Rahman Rasel, Abrar Ahamed Habibullah, Md Moyazzem Hossain

**Affiliations:** 1 Department of Statistics, Jahangirnagar University, Savar, Dhaka 1342, Bangladesh; 2 Department of Civil Engineering, University of Asia Pacific, Dhaka1205, Bangladesh; 3 Department of Electrical and Computer Engineering, North South University, Bashundhara, Dhaka1229, Bangladesh

**Keywords:** Bangladesh, mobile game addiction, ordinal regression, university students

## Abstract

**Background:**

Nowadays, the youth are more engaging with their more advanced smartphones having high-quality graphics and gaming features. However, existing literature depicts that adolescents suffer from several forms of psychological problems including mental health, depression, loneliness, insomnia and low self-control due to mobile game addiction. Therefore, this study aims to find the prevalence and motivating factors for mobile game addiction among university students of Bangladesh.

**Methods:**

A cross-sectional survey was carried out to collect the required information from 1125 students of three universities in Bangladesh. Descriptive statistics, χ^2^ test and ordinal regression model are employed to meet the objective of this study.

**Results:**

The findings reveal that male students are more likely to show addictive behaviours than their counterparts in the context of mobile game addiction. The results depict that loneliness, duration of using smartphones and playing mobile games, and source of entertainment are the main cause of mobile addiction. Also, more than half of the respondents (54.3%) are severely addicted to mobile games who were influenced by friends and YouTube gamers to play games. Moreover, students are suffering from several physical problems such as headaches, eye discomfort, blurry vision and ear discomfort.

**Conclusion:**

Considering the findings of this paper, the authors suggest that the authorities should consider this immediately and arrange a positive entertainment environment to prevent students from mobile games. Furthermore, it is necessary to encourage students to participate in sports or other extracurricular activities that may be helpful to lessen mobile game addiction among students in Bangladesh.

## Introduction

The current era is technology-dependent and over the last decades, technology has improved tremendously. Almost every sector is trying to absorb the recent technology for completing their tasks smoothly as well as accurately within a minimum time in every corner of the world. Youth are now much more engaged with their more sophisticated smartphones, which have high-quality graphics and gaming features. Among the broad range of activities, gaming is one of the activities using modern generation smartphones in addition to phone calls and texting. Recently, more developed and advanced smartphones offer high-quality graphics and gaming features to users which attract them to play more time. Most of the games can be downloaded for free which is known as ‘freemium games’ but need to pay for extra features (Su *et al*., 2016). A study pointed out that new generation mobile games are more challenging and players choose to play challenging games (Balakrishnan and Griffiths, 2019). In recent years, Massively Multiuser Online Role-Playing Games (MMORPG) have been the most popular and challenging games (Achterbosch *et al*., 2008; Scott and Porter-Armstrong, 2013; Sourmelis *et al*., 2017; Raith *et al*., 2021). Previous studies illustrated that MMORPGs are more addictive to men than women due to their interactive, collaborative and competitive nature (Liu and Peng, 2009; Barnett and Coulson, 2010; Laconi *et al*., 2017).

According to Donati *et al*. (2015) game genres play a vital role as a risk factor for game addiction (Donati *et al*., 2015). Mobile game addiction is a part of Internet gaming disorder (Laconi *et al*., 2017). Researchers from all over the world have performed studies to determine the motivations for this addiction. Sherry *et al*. (2006) investigated that 68% of adolescents play mobile games as their weekly entertainment (Sherry *et al*., 2006). Most of the researchers mentioned two major factors of addiction – Escapism and Advancement. Some young people are too shy or scanty on social communication and low self-esteem that game playing or the virtual world becomes a substitute for real-life interaction (Lo *et al*., 2005; Wan and Chiou, 2006; Yee, 2006*b*; Hussauin and Griffiths, 2009). Moreover, they play video game to avoid, forget, or escape from real-life stress and problem (Yee, 2006*a*). Another key motive is the need for advancement that is trying to become more powerful, advance, achieve a lot, win and challenge others (Wan and Chiou, 2006; Yee, 2006*b*, 2006*a*). In the last few years, negative consequences due to mobile game addiction are rapidly studied and warned by the researchers. Researchers pointed out that adolescents suffer from poorer mental health, cognitive functioning, social anxiety, depression, loneliness, stress, lack of sleep, personality disorder and low self-control due to mobile game addiction (Ferguson *et al*., 2011; Gentile *et al*., 2011; Cheever *et al*., 2014; Jeong *et al*., 2016). Studies also show that people become less interactive with their family, friends, society and also get detached from their studies and other physical activities because of mobile game addiction (Stockdale and Coyne, 2018). Though there are enough negative consequences, Samaha and Hawi (2016) found that there is no relationship between mobile game addiction and academic performance (Samaha and Hawi, 2016). Fabito *et al*. (2018) also studied that mobile game addiction among tertiary students does not have any relationship with their academic performance (Fabito *et al*., 2018).

Very few studies were conducted about mobile game addiction among children and adolescents but there is no study conducted before about this type of addiction among university students in Bangladesh. Though a study investigated parent–child relationship and parenting as a causal factor for digital game addiction among children aged 5–18 years old in the north-western part of Bangladesh (Keya *et al*., 2020), there is still a lack of information about the motivations of addiction, addictive behaviour and its negative impacts among university students in Bangladesh. Aiming to address the lack, the objective of this study is to determine the relationship between mobile game addiction and academic performances among tertiary students in Bangladesh. This study also finds the motivating factors for mobile game addiction, addictive behaviours and its negative consequences among university student perspectives of Bangladesh.

This paper is organized as follows: Methods section contains participants, design and data collection procedure, and statistical techniques used in this study. The Results and Discussion sections describe and compare findings with relative other findings.

## Methods

### Participants

This explorative study consisted of 1125 students from three universities in Bangladesh namely Jahangirnagar University (*n* = 346), University of Asia Pacific (*n* = 418) and North South University (*n* = 361). Initially, a total of 1169 responses were collected, among them 44 responses were incomplete and they are excluded from the final data set. The final sample consisted of 1125 responses, and among them, 69.1% (*n* = 777) and 30.9% (*n* = 348) of the respondents were male and female, respectively. Age of the respondents ranging from 18 to 28 years (mean: 22.44 years, standard deviation: 1.91 years).

### Design and procedure

The online survey was conducted among 1125 respondents from the selected three universities in Bangladesh. In this current pandemic situation, it is not feasible to do a face-to-face survey and that is why the data are collected online via the link of the designed google form. Prior to participating in this survey, the authors inform the respondents about the purpose of this study and ensure that the information they provide would be kept confidential and oral consent is taken. Respondents who gave consent to participate in this survey were then sent a link to the questionnaire and accompanying instructions to complete it. Despite the fact that this research is not related to human trials, it was carried out in accordance with the Declaration of Helsinki ethical standard. The survey was conducted over a 1-month period from 23 October 2020 to 27 November 2020.

The online survey questionnaire was partitioned into two sections: (a) socio-demographic and (b) Lemmens' game addiction scale (Lemmens *et al*., 2009). The socio-demographic part includes sex, age, university name, social relationship, physical sports involvement, resource availability and time spent on playing. To evaluate the addiction level, Lemmens *et al*. (2009) developed and validated the game addiction scale. This game addiction scale consisted of 21 questionnaires that constitute seven criteria and three items of questions were provided for each criterion. All questions were answered using a five-point Likert scale (1–5) that is: (i) Never, (ii) Rarely, (iii) Sometimes, (iv) Often, (v) Very often. Descriptions of these seven criteria are given in Table 1.

**Table 1.** Seven criteria for the scale of mobile game addiction

Criteria name	Descriptions
1. Salience	Playing mobile games becoming most important and dominant on thoughts, affection and attitude
2. Tolerance	Play mobile games for satisfaction
3. Mood modification	Game is used as transforming mechanism of bad mood to good mood
4. Relapse	While reducing playtime, tendency to restore earlier excessive playing pattern
5. Withdrawal	Psychological discomfort or unpleasant when playtime is decreased or discontinued
6. Conflict	Interpersonal conflict due to excessive gaming or playtime
7. Problems	Problems in workplace, schools or social place due to excessive gaming

### Statistical methods

Quantitative research approach was applied in this explorative study. The cross-tabulation was carried out for descriptive analysis along with the χ^2^ test and Goodman and Kruskal's *γ* (*G*) (Goodman and Kruskal, 1954), for making comparisons among variables and ascertain the significant relationship between the considered variables and the level of mobile game addiction. Cronbach's *α* has been reported to check the internal consistency of the variables in this study. Here, the reliability of the data was checked using Cronbach's *α* developed by Lee Cronbach in 1951 (Cronbach, 1951) which lies between 0 and 1 and the value close to 1 provides more reliability (Nunnally and Bernstein, 1994). The acceptable value of Cronbach's *α* is 0.70 (Nunnally, 1978; Zikmund, 1999). The Cronbach's *α* can be defined as 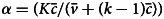, where *K* is the number of components (*K* items), 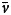 is the average variance of each component (item) and 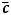 is the average of all covariances between the components across the current sample of persons, that is, without including the variances of each component. Finally, as the outcome variable is classified according to their order of magnitude, ordinal logistic regression (OLR) analysis has been conducted to examine the influences of targeted variable on mobile game addiction. Let *Y* be an ordinal outcome with *j*categories. Then *P*(*Y* ≤ *j*) is the cumulative probability of *Y*less than or equal to a specific category *j* = 1, 2, …, *J* − 1. The odds of being less than or equal to a particular category can be defined as, *P*(*Y* ≤ *j*)/*P*(*Y* > *j*) for *j* = 1, 2, …, *J* − 1 since *P*(*Y* > *J*) = 0 and dividing by zero is undefined. The log odds is also known as the logit, so that log (*P*(*Y* ≤ *j*)/*P*(*Y* > *j*)) = logit(*P*(*Y* ≤ *j*). The general form of the OLR model can be written as,
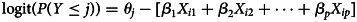
where, logit() is the link function, *θ*_*j*_ is the threshold for the *j*th category, *p* is the number of regression coefficients, *X*_*i*1_, *X*_*i*2_, …, *X*_*ip*_ are the values of the predictors for the *i*th case and *β*_1_, *β*_2_, …, *β*_*p*_ are regression coefficients (McCullagh and Nelder, 1989; Harrell, 2015).

In OLR, several link functions, i.e. cauchit, complementary 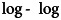, logit, negative 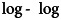 and probit were used in several research (Liao, 1994; Javali and Pandit, 2010; Agresti, 2013; Fernández-Navarro, 2017; Smith *et al*., 2019; Singh *et al*., 2020). Among these link functions, negative log-log ( − log ( − log (*y*))) has been used in this study since lower categories are more probable to building up model. For better understanding and interpretations, odds ratio has also been calculated. All the analyses were performed using SPSS 25.

## Results

Reliability analysis was conducted to check the internal consistency of the variables. The value of Cronbach's *α* is 0.852 for all variables considered in this study and 0.921 for the 21-item game addiction scale indicates excellent internal consistency. The cross-tabulation was carried out for descriptive analysis and ascertains the significant relationship between the considered variables and the level of mobile game addiction and the results are presented in Table 2. The significance of the association is tested using the χ^2^ test and Goodman and Kruskal's *γ*. The level of mobile game addiction is evaluated using the guidelines of Lemmens' GAS-21 questionnaires (Lemmens *et al*., 2009) and categorized as mild, moderate and severe. Gender has a significant relationship with the level of mobile game addiction (*p* value < 0.001). Results reveal that among all the participants of this study, the highest percentage (56.4%) of males are moderately addicted to mobile games and 15.4% of male students are severely addicted. Female students have a lower level of mobile game addiction compared to their counterparts in moderate and severe levels; however, the female students have more mild level addiction than male students. It is observed that the age of the respondent is significantly associated with mobile game addiction (*p* value < 0.001). Results indicate an increasing addiction level of mobile game addiction with increasing the age of university students. Surprisingly, it is found that students whose age is 25 years or more are at higher risk, i.e. more than half of the respondents (51.9%) of students whose age is 25 years or more expose to severe addiction to mobile games. More than 40% of students whose age is less than 22 years have mild (41.6%) and moderate (44.7%) levels of mobile game addiction whereas only 13.7% of students are severely addicted to a mobile game. Most of the students (61.9%) whose age is between 22 and 25 years are exposed to a moderate level of addiction to mobile games but 29.8% and 8.3% of students are mildly and severely addicted, respectively, to mobile games (Table 2).

**Table 2.** Per cent distribution of the label of mobile game addiction by selected characteristics

Characteristics	Level of mobile game addiction (%)			*p* value of the χ^2^	*p* value of Goodman and Kruskal's *γ*
	Mild	Moderate	Severe		
Sex					
Male	28.2	56.4	15.4	<0.001	<0.001
Female	49.1	39.9	11.0		
Age (years)					
<22	41.6	44.7	13.7	<0.001	<0.001
22–25	29.8	61.9	8.3		
25+	17.3	30.9	51.9		
University					
Jahangirnagar University	42.8	43.4	13.9	<0.001	<0.001
North South University	23	62	15		
University of Asia Pacific	38	48.6	13.4		
Way of getting smartphone					
From family	36.6	51.2	12.2	0.002	0.001
Self-income	28.3	51.6	20.2		
Smartphone use starting age					
8–12 years	8.3	75	16.7	<0.001	<0.001
13–17 years	29	55.5	15.5		
18 and more	46.6	42.2	11.2		
Whether involved in indoor/outdoor games or not					
Yes	26.8	60.7	12.6	<0.001	<0.001
No	49.4	33.8	16.8		
Influenced by					
Friends	28.9	56.2	14.9	<0.001	<0.001
Family members	31.3	68.8	0.0		
Yourself	32.2	56.6	11.2		
Friend and yourself	17.2	73.9	8.9		
Friend, yourself and YouTube gamers	10.4	56.3	33.3		
Friend and YouTube gamers	6.5	39.1	54.3		
None	68.9	25.1	6.0		
Yourself and YouTube gamers	24.1	58.6	17.2		
Main source of entertainment					
Yes	23.3	61.3	15.4	<0.001	<0.001
No	55.9	32.7	11.5		
Duration of play mobile games					
<2 h	41.5	50.5	8.0	<0.001	<0.001
2–4 h	15.2	60.7	24.1		
4 h and more	5.5	35.6	58.9		
Time of playing mobile game					
Day time	49.0	45.8	5.2	<0.001	<0.001
Night time	34.3	54.8	10.9		
Both day and night time	18	50	32		
Health problem					
Blurry vision	25	50	25	<0.001	<0.001
Blurry vision and eye discomfort	11.7	76.7	11.7		
Blurry vision, headache and eye discomfort	29.9	42.5	27.6		
Eye discomfort	31.3	61.4	7.3		
Headache	40.0	48.5	11.5		
Headache and ear discomfort	19.4	48.6	32.0		
None	50.3	44.9	4.8		

The result depicts that students who started to use a smartphone at a very early age have been exposed to higher addiction significantly (*p* value < 0.001). About one-third of the respondents have moderate and 16.7% of students have a severe level of addiction to mobile games who started to use smartphones at the age of 8 to 12 years, whereas only 15.5% and 11.2% of students are severely addicted to mobile games who started to using smartphone at the age of 13 to 17 years and 18+ years respectively. Moreover, 16.8% of students who are severely addicted to mobile games are not involved in any indoor or outdoor games which is slightly higher than those (12.6%) who involve in any kind of indoor or outdoor games (*p* value < 0.001). Findings also suggest that more than half of the respondents (54.3%) are severely addicted to mobile games who are influenced by their friends and various YouTube gamers who suggest playing mobile games. Also, 68.9% of students who are not influenced by anyone to play the games are in a safe zone because they have a mild level of addiction to playing mobile games (*p* value < 0.001). The moderate level of addiction is double for those who play mobile games as the main source of entertainment than their counterparts. The prevalence of severely addicted is also slightly higher among the students who play mobile games as the main source of entertainment than those who do not play mobile games as the main source of entertainment (15.4% *v.* 11.5%). Duration of playing a mobile game is highly significantly associated with mobile game addiction (*p* value < 0.001). Only 8% of students are exposed to severe addiction levels when they play the mobile game for less than 2 h a day. Approximately, one out of five students is exposed to severe addiction levels when they play mobile games for 2–4 h daily. It is alarming that the prevalence of severely addicted to mobile games is very high (58.9%) among the students who play mobile games for more than 4 h a day. The result also shows that 32% of students are severely addicted to mobile games who choose to play a mobile game both day and night, whereas only 5.2% of students are severely addicted who choose to play at day time only and 10.9% of students are severely addicted who choose to play at night time only (*p* value < 0.001). Students report that they are facing some health problems such as blurry vision, eye discomfort, headache and so on and the findings depict that different health problems faced by the participants are significantly associated with the level of mobile game addiction (*p* value < 0.001). Among students who suffer from both headache and ear discomfort, 48.6% and 32% of students are moderately and severely addicted to mobile games, respectively. Furthermore, among the students who suffer from blurry vision, headache and eye discomfort, 42.5% and 27.6% of students are moderately and severely addicted to mobile games, respectively (Table 2).

**Fig. 1.**
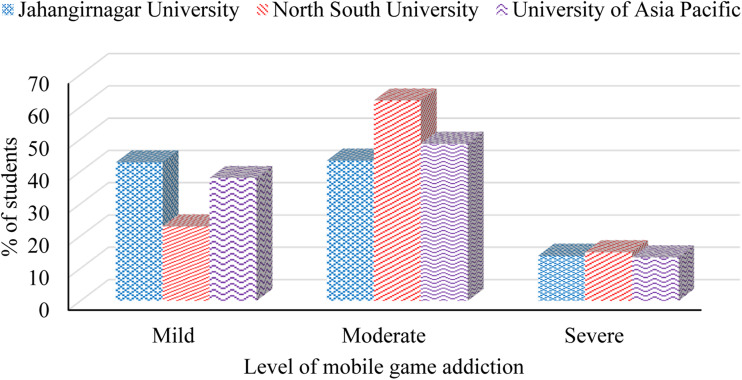
Level of mobile game addiction among students by university.

Results indicate that 13.9, 15 and 13.4% of participants who are currently studying at Jahangirnagar, North South and Asia Pacific University, respectively, are severely addicted to mobile games. Findings reveal that more than 20% of students from North South University have a mild level of mobile game addiction while the prevalence of mild level addiction is almost double among the students of the other two universities. A slightly higher addiction at moderate and severe levels is observed among the students of North South University compared to the students of the other two universities considered in this study (Fig. 1).

### Ordinal logistic regression

For the logistic regression model, we do not have the option of checking multicollinearity in SPSS. However, we fit multiple linear regression prior to performing OLR for checking the multicollinearity considering variance inflation factor (VIF). The value of VIF < 2.5 for the set of independent variables ensures the absence of multicollinearity. Moreover, a study pointed out that multicollinearity is no more a problem to fit the logistic regression model (Senaviratna and Cooray, 2019). The results of the OLR model are presented in Table 3. The target variable for this analysis is the levels of mobile game addiction (mild, moderate and severe). The result reveals that male students have an approximately 1.3 times (OR 1.29) higher chance of being severely addicted to mobile games compared with female students. The students of Jahangirnagar University and North South University have 1.2 and 1.4 times, respectively, more likelihood of being severely addicted to mobile games compared with the students of the University of Asia Pacific. The result also reveals that loneliness is significantly associated with mobile game addiction among university students. Students are exposed to lower addiction levels when they have companionship most of the time compared to those who are agreed with feeling isolated. An increasing odds associated with the levels of the response of playing the mobile game when feeling isolated indicates that the more they feel isolated, the chance of being severely addicted increases. This study found that students who do not want to be a master player and just played for self-enjoyment are exposed to lower addiction levels. Wish to become the master of the game does not have a significant relationship with mobile game addiction. Finding also reveals that to be entertained by mobile games is significantly associated with mobile game addiction. Those who have chosen mobile games as the main source of entertainment have about 1.3 times higher chance of being severely addicted compared to those who have not chosen mobile games as the main source of their entertainment.

**Table 3.** Results of ordinal regression model

Variable	Level	Coefficient	Odds ratio	*p* value	95% CI of coefficient	
					Lower bound	Upper bound
Addiction	Mild	−3.791	–	0.0001	−5.295	−2.288
	Moderate	−1.673	–	0.028	−3.163	−0.183
Age		0.055	1.057	0.027	0.006	0.103
Smartphone use starting age		−0.071	0.932	0.000	−0.111	−0.031
Sex	Male	0.255	1.290	0.008	0.068	0.443
	Female (Ref.)	–	–	–	–	–
University	Jahangirnagar University	0.218	1.244	0.030	0.021	0.416
	North South University	0.337	1.401	0.007	0.094	0.581
	University of Asia Pacific (Ref.)	–	–	–	–	–
Way of getting smartphone	From family	−0.131	0.877	0.196	−0.331	0.068
	Self-income (Ref.)	–	–	–	–	–
Playing mobile game is more comfortable than hang out with friends	Strongly disagree	−0.195	0.823	0.571	−0.870	0.480
	Disagree	−0.080	0.923	0.811	−0.737	0.577
	Neither agree nor disagree	0.191	1.210	0.580	−0.485	0.866
	Agree	0.130	1.139	0.711	−0.555	0.815
	Strongly agree (Ref.)	–	–	–	–	–
Feeling more comfortable in a virtual game environment than in outdoor game/social relationship	Strongly disagree	0.092	1.096	0.752	−0.477	0.660
	Disagree	−0.210	0.811	0.456	−0.761	0.342
	Neither agree nor disagree	−0.172	0.842	0.559	−0.751	0.406
	Agree	−0.162	0.850	0.580	−0.736	0.412
	Strongly agree (Ref.)	–	–	–	–	–
Play mobile games when feeling isolated	Strongly disagree	−0.938	0.391	<0.001	−1.430	−0.446
	Disagree	−0.856	0.425	<0.001	−1.238	−0.475
	Neither agree nor disagree	−0.540	0.583	0.003	−0.900	−0.179
	Agree	−0.491	0.612	0.003	−0.818	−0.163
	Strongly agree (Ref.)	–	–	–	–	–
Playing mobile games to cope with the new game/updated version of game	Strongly disagree	−0.454	0.635	0.144	−1.062	0.154
	Disagree	−0.118	0.889	0.676	−0.671	0.436
	Neither agree nor disagree	−0.455	0.635	0.101	−0.998	0.089
	Agree	−0.289	0.749	0.284	−0.818	0.239
	Strongly agree (Ref.)	–	–	–	–	–
Playing mobile games for a long time to become a master of game	Strongly disagree	−0.900	0.407	0.002	−1.463	−0.338
	Disagree	−0.542	0.582	0.038	−1.055	−0.029
	Neither agree nor disagree	−0.067	0.935	0.804	−0.593	0.460
	Agree	0.133	1.142	0.610	−0.379	0.645
	Strongly agree (Ref.)	–	–	–	–	–
Playing mobile games for the main reason of entertainment	Yes	0.246	1.279	0.007	0.068	0.425
	No (Ref.)	–	–	–	–	–
Duration of playing mobile games	<1 h	−1.448	0.235	<0.001	−1.997	−0.898
	1–2 h	−1.039	0.354	<0.001	−1.587	−0.491
	3–4 h	−1.065	0.345	<0.001	−1.635	−0.494
	4–5 h	−0.723	0.485	0.020	−1.334	−0.112
	5–6 h	−0.188	0.829	0.651	−1.003	0.627
	6–8 h (Ref.)	–	–	–	–	–
Playtime	6:00–12:00 h	−0.054	0.947	0.771	−0.416	0.309
	12:00—18:00 h	−0.423	0.655	0.002	−0.692	−0.153
	18:00–0:00 h	−0.312	0.732	0.008	−0.542	−0.083
	0:00–6:00 h	−0.206	0.812	0.127	−0.471	0.059
	12:00–18:00 and 0:00–6:00 h (Ref.)	–	–	–	–	–
Negative impact on academic performance due to playing excessive mobile game	Strongly disagree	−0.962	0.382	<0.001	−1.343	−0.581
	Disagree	−0.774	0.461	<0.001	−1.116	−0.432
	Neither agree nor disagree	−0.454	0.635	0.010	−0.800	−0.108
	Agree	−0.079	0.924	0.652	−0.422	0.264
	Strongly agree (Ref.)	–	–	–	–	–
Model fitting information						
Cox and Snell *R*^2^		0.68				
Omnibus Tests of Model Coefficients		516.38***				
Cohen's *κ*		0.43***				
Sensitivity		72.16				
Specificity		65.99				
Test of parallel lines						
*χ*^2^		127.50***				

Ref., reference category; CI, confidence interval; *p < 0.05, **p < 0.01, ***p < 0.001.

Moreover, the duration of playing a mobile game is considered an important factor of mobile game addiction levels. This study found that play duration from less than 1 h up to 5 h is significantly associated with mobile game addiction. Students who play mobile games in this time range expose to a lower level of addiction. Addiction level increases as the duration of playing mobile games increases. Findings also reveal that those who choose to play mobile games between 12:00 and 0:00 h have been exposed to addiction. It is found that only two time slots of playing mobile games (12:00 to 18:00 h and 18:00 to 0:00 h) in a day are significantly associated with mobile games addiction. Moreover, results depict that students who strongly disagree with the statement that playing excessive mobile games has a negative impact on academic performance have a lower chance of addiction compared with the students who strongly agree that excessively playing mobile games has an adverse impact on academic performance. The findings reveal that there is no significant relationship between addiction or playing excessive mobile games and academic performances among university students (Table 3).

It is expected that students who expose addictive behaviours may feel more comfortable with playing mobile games or the virtual environment most of the time than hang out with friends or social relationships. However, this study found no significant relationship between addiction and feel comfortable in playing mobile games or comfortable in the virtual environment than time passing with friends and social relationships. We also expected that active gamers always have a tendency to play new games that lead to their addiction. But results show that there is no significant relationship between addiction and intention to play new games. In this study, the way of getting or source of the smartphone, that is, how a student gets his first smartphone and there are two possible answers considered such as ‘from family’ or ‘self-income’. In most cases, those who are given a smartphone from the family usually get it at an early age. So they have been familiar with playing these mobile games very earlier and gradually develop their addiction. We expect a significant relationship between addiction and get a smartphone from family compared to self-income. However, the findings of this study reveal that there is no significant relationship between addiction and the source of getting a smartphone. The value of the χ^2^ test for testing the parallel lines, i.e. the location parameters (slope coefficients) are the same across response categories, is rejected (χ^2^: 127.5, *p* value < 0.001). Moreover, the results of Omnibus Tests of Model Coefficients and Cox and Snell *R*^2^ indicate that the fitted model is good. The value of Cohen's *κ* statistic for our data set is 0.43 which depicts that the fitted model has a ‘moderate’ discriminative power. Furthermore, the sensitivity and specificity are 72.16% and 65.99%, respectively (Table 3).

**Fig. 2.**
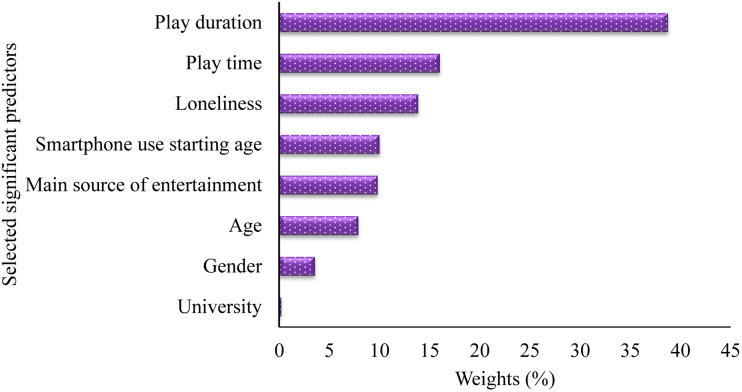
Selected significant predictors responsible for influencing mobile game addiction among students.

The ordering of the significant factors in terms of their weights to predict the outcome was made possible by ranking the relatively important scores. The importance score was produced to assist us in identifying the key predictor variables that are most likely to influence the outcome. The findings reveal that the duration of playing a mobile game is the most significant predictor, followed by playtime, loneliness, smartphone use starting age, consider as a main source of entertainment, age, sex and name of the university (Fig. 2).

## Discussion

The aim of this paper was to find out the prevalence of mobile game addiction among university students in Bangladesh. Attempts have been made to investigate the motivating factors for this addiction and inquired about its adverse consequences. Our result showed that male students are more likely to show addictive behaviours than female students in the context of mobile game addiction. This study has confirmed a correlation between an increasing mobile game addiction with increasing age among university students. Surprisingly, among the Bangladeshi students, those who are nearing the end of their academic studies session are more addicted than those who are just admitted into the university. Our findings have revealed an interesting link between mobile game addiction and smartphone usage age. Students who have been using a smartphone for at least 10 years or more time have developed higher addiction. If this duration increases, a person gradually develops his/her addiction. Students who are very familiar with smartphones at a very early age are exposed to higher addiction than those who had started using a smartphone when they were adults. This finding indicates smartphone usage age as one of the motivating factors for developing mobile addiction. We found strong evidence that loneliness is also the causal factor that can drive one to addiction. Loneliness can cause several negative consequences in human lives. Starting from different types of addiction (mobile game addiction is one of them), it is also responsible for deadly acts such as attempts to suicide (Stravynski and Boyer, 2001; Chang *et al*., 2019; Abi-Jaoude *et al*., 2020). We should be aware now to be saved from the curse of loneliness. Not only students, everyone should help to bring out their family members, friends and classmates from loneliness. When students are detached from their friends, family and social interaction, they are exposed to higher addiction to mobile games. These findings supported by other studies where the researchers found that people get less interactive with their family, friends, society and also detached from their studies and other physical activities are one of the main reasons for mobile game addiction (Wang and Zhu, 2011; Stockdale and Coyne, 2018).

This study result measures different addiction levels for both types of students who are involved in any indoor or outdoor games and who are not. There is clear evidence that students who are not involved in any indoor or outdoor games are highly addicted to mobile games than others. This study states this involvement as one of the major motivating factors for mobile game addiction. Entertainment is another factor that gradually develops an addiction. Results found that 65.2% of university students (male = 72.7% and female = 48.3%) of Bangladesh play mobile games as their main source of entertainment. Results also indicate that students have a higher chance of being severely addicted to mobile games who play it as the main source of their entertainment. These findings are similar to the findings of Sherry *et al*. (2006) who found that 68% of adolescents play mobile games as their weekly entertainment (Sherry *et al*., 2006). Result confirms that being influenced by others is another major motivating factor that has a strong relationship with severe addiction levels. Shocking findings are that more than half of the respondents (54.3%) are severely addicted to mobile games who were influenced by friends and YouTube gamers to play. But at the same time, results also showed that students who are not influenced by others are in the safe zone. The need for advancement does not have a significant effect on mobile game addiction among university students in Bangladesh. Results depict that students are not intended to play to become a master of the game or achieve a lot, they just do it for self-entertainment. Though there are several studies that argue the need for advancement plays a vital role as a motivating factor for developing mobile game addiction (Wan and Chiou, 2006; Yee, 2006*a*, 2006b), our study findings do not support that at all. Our result provided strong evidence that play duration is another key factor for developing mobile game addiction. The longer time they play in a day, the higher addiction they are exposed to. The results also reveal that one out of five students who choose to play mobile games between 2 and 4 h in a day are exposed to severe addiction. More shocking findings are that almost six students out of 10 (58.9%) are severely addicted to playing more than 4 h a day. The results mentioned a specific time interval (12:00 to 0:00 h) in a day when most of the gamers choose to play and mobile game addiction is significantly associated with this time interval.

The results show that playing mobile games most of the time (both day and night) between this time interval indicates a very higher risk of being severely addicted to the mobile game. One of the major objectives of this study was to determine the relationship between mobile game addiction and academic performance. Our results provided strong evidence that there is no relationship between mobile game addiction and academic performance. This is the first study that revealed that mobile game addiction does not have an inverse impact on academic performances among university students in Bangladesh. Our results are strongly supported by the findings of Fabito *et al*. (2018) who studied in earlier research that mobile game addiction among tertiary students does not have any relationship with their academic performance (Fabito *et al*., 2018). In another study, Samaha and Hawi (2016) also found that there is no relationship between mobile game addiction and academic performance which also generalizes our findings (Samaha and Hawi, 2016). The results of this study revealed some major health problems which are associated with severe addiction level. Moreover, 32% of severely addicted students suffer from headaches and ear discomfort. Also, 27.6% of severely addicted students suffer from blurry vision and eye discomfort along with headaches. Researchers argue that addicted gamers substitute their real-life interaction or social relationship with the virtual environment (Lo *et al*., 2005; Wan and Chiou, 2006; Hussauin and Griffiths, 2009). But this study did not find enough evidence to establish this opinion. Also, this study did not find enough evidence to establish eagerness to play a new game is a causal factor for developing addiction levels. It has been confirmed that the source of a smartphone is not associated with mobile game addiction.

## Limitations and future research

Though the current study contributes to the literature providing many insights information and the present situation of Bangladeshi university students in the context of mobile game addiction, it is not far beyond the limitations. This study considers three university students only. If we could include more universities in our study sample, the result would be more accurate. This study was conducted through an online survey; as a result, participants could skip few questions. To gather much information via face-to-face survey or interview method will be more efficient. Since GAS-21 is only an addiction measurement scale, this study does not provide a psychological condition of gamers. To evaluate the post psychometric condition of addictive gamers, a future study should be carried out using psychological tests along with a game addiction test. Moreover, this study does not consider effect size for making a comparison between groups. In the future study, the researcher may use snowball sampling to acquire active gamers or addictive gamers as a sample so more insights into addictive behaviour could be revealed and focussed on the effect size to compare the groups. Also, we will try to conduct a study considering DASS-21, SHAI, AIS scales and find the linkage among them. This study encourages researchers to study the prevalence rate of mobile game addiction among school and college students in Bangladesh.

## Conclusion

The current study clearly enunciates some major motivating factors for mobile game addiction and relationships among university students in Bangladesh. Firstly, this study confirms that loneliness encourages addiction to mobile games. Secondly, this study revealed that about 65% of students chose to play mobile games as their medium of entertainment. Regrettably, the medium of entertainment should not be limited to playing mobile games where there are many negative consequences. Authorities should take this into consideration immediately and arrange a positive entertainment environment and make the students interested in it. Thirdly, not engaging in any physical indoor or outdoor games encourages one to develop an addiction. Engagement in sports or other extracurricular activities may reduce loneliness and thus reduces addiction. Each university has several extracurricular clubs; there are vast opportunities to involve themselves in various activities. Just need to give them a little more extra encouragement to participate. Fourthly, mobile game addiction increases with age. Physical impacts such as headache, eye discomfort, blurry vision and ear discomfort are major findings of this study. Responsible authorities should immediately take into account these forms of addiction and take the necessary step to get rid of this addiction.
